# EGCG modulates PKD1 and ferroptosis to promote recovery in ST rats

**DOI:** 10.1515/tnsci-2020-0119

**Published:** 2020-05-29

**Authors:** Jianjun Wang, Ying Chen, Long Chen, Yanzhi Duan, Xuejun Kuang, Zhao Peng, Conghui Li, Yuanhao Li, Yang Xiao, Hao Jin, Quandan Tan, Shaofeng Zhang, Bopei Zhu, Yinjuan Tang

**Affiliations:** Affiliated Hospital, Xiangnan University, Chenzhou 423000, Hunan Province, China; Department of Clinical, Xiangnan University, Chenzhou 423000, Hunan Province, China; Jilong Union School of Hengnan County, Hengyang 421000, Hunan Province, China; Department of Basic Medical Sciences, Xiangnan University, Chenzhou 423000, Hunan Province, China

**Keywords:** spinal cord injury, (−)-epigallocatechin-3-gallate, cerebellar granule neurons, ferroptosis, oxidative stress

## Abstract

**Background:**

Spinal cord injury (SCI) causes devastating loss of function and neuronal death without effective treatment. (−)-Epigallocatechin-3-gallate (EGCG) has antioxidant properties and plays an essential role in the nervous system. However, the underlying mechanism by which EGCG promotes neuronal survival and functional recovery in complete spinal cord transection (ST) remains unclear.

**Methods:**

In the present study, we established primary cerebellar granule neurons (CGNs) and a T10 ST rat model to investigate the antioxidant effects of EGCG via its modulation of protein kinase D1 (PKD1) phosphorylation and inhibition of ferroptosis.

**Results:**

We revealed that EGCG significantly increased the cell survival rate of CGNs and PKD1 phosphorylation levels in comparison to the vehicle control, with a maximal effect observed at 50 µM. EGCG upregulated PKD1 phosphorylation levels and inhibited ferroptosis to reduce the cell death of CGNs under oxidative stress and to promote functional recovery and ERK phosphorylation in rats following complete ST.

**Conclusion:**

Together, these results lay the foundation for EGCG as a novel strategy for the treatment of SCI related to PKD1 phosphorylation and ferroptosis.

## 1 Background

Many studies have reported that severe spinal cord injury (SCI) leads to neuronal death and axonal loss in the local microenvironment, eventually resulting in a devastating loss of function [1,2]. Various intrinsic and extrinsic mechanisms have been investigated to promote developmentally related and injury-induced changes that limit neuronal survival in the adult central nervous system [3,4].

Many of the beneficial effects of green tea on the nervous system were attributed to its abundant catechin. (−)-Epigallocatechin-3-gallate (EGCG), the major catechin found in green tea (*Camellia sinensis*), has been extensively investigated as the predominant active polyphenol and a promising therapeutic agent for the treatment of chronic inflammation and oxidative damage-related diseases [5,6,7]. EGCG also inhibits the TNF-α-activated nuclear factor-kappa B (NF-kB) pathway and enhances the nuclear factor E2-related factor 2 protein levels in macrophages [8]. EGCG was verified to protect against liver injury via its antioxidant effects [9]. EGCG targeting HO-1 reduces contrast-induced kidney damage through antioxidative stress pathways [10]. Several experimental studies have shown that EGCG can provide neuroprotection against brain injury, SCI, and sciatic nerve injury [11,12]. These benefits are mainly due to free radical scavenging or the antioxidant and anti-apoptotic properties of EGCG [13,14]. However, the underlying mechanisms remain to be investigated.

Protein kinase D1 (PKD1), also called protein kinase Cµ [15], is a serine/threonine protein kinase that is different from PKC family members owing to its special structural, regulatory, and enzymatic properties [16]. Moreover, PKD1 can function not only in a normal state but also in a diseased state [15,17,18]. PKD1 is increasingly implicated in modulating a diverse range of functions within the cell, including signal transduction pathways, cell proliferation, angiogenesis, invasion, motility, survival, and apoptosis [17,19,20,21,22,23]. It has been shown that activation of PKD1 induced by oxidative stress can, in turn, activate the transcription factor NF-κB to protect injured cells from cell death following oxidative stress-induced lesions [24]. Interestingly, the neuronal damage caused by oxidative stress has been shown to be involved in SCI [25,26].

Ferroptosis was recently identified as an iron-dependent novel cell death mechanism [27,28,29] with morphological, genetic, and mechanistic differences from traditional regulated cell death pathways, including necrosis, apoptosis, and autophagy [27,28,30]. Apoptosis is a noninflammatory process, and the process of iron-induced cell death is often accompanied by inflammatory manifestations. Ferroptosis is a result of failure of membrane lipid repair, leading to the accumulation of reactive oxygen species (ROS) on membrane lipids [31,32,33] and eventually leading to cell death. Ferroptosis requires the simultaneous depletion of glutathione (GSH) or inactivation of GSH-dependent antioxidant enzyme glutathione peroxidase 4 (GPX4) and the incorporation of oxidizable polyunsaturated fatty acids into phospholipids [34], and the process of iron-dependent cell death is accompanied by inflammatory manifestations.

Therefore, the aim of this study was to investigate the antioxidant effects of EGCG on promoting neuronal survival and functional recovery after spinal cord transection (ST), focusing on its anti-ferroptotic properties and its modulatory effect on PKD1 phosphorylation.

### 1.1 Primary culture of cerebellar granule neurons (CGNs) and treatments

The primary culture of CGNs was performed according to previous studies with minor modifications [35,36,37]. Briefly, cerebellum from P7 rats was dissected. The tissues were kept on ice in Ca^2+^/Mg^2+^-free Hank’s balanced salt solution, followed by digestion with 0.125% trypsin at 37°C in a humidified 5% CO_2_ atmosphere for 30 min followed by trituration and seeding into culture wells. After 4 h in culture with complete Dulbecco's modification of Eagle's medium (DMEM), the medium was replaced with Neurobasal-A (Life Technologies) culture medium supplemented with 2% B27 (Life Technologies) and 1% penicillin/streptomycin mixture (Solarbio Biotech Corp).

To test the effect of EGCG on the CGNs, cells were subjected to two different treatments: (i) CGNs were treated with Neurobasal-A medium containing 0, 0.5, 5, 50, or 500 µM EGCG for 48 h, followed by MTT assay; (ii) CGNs were exposed to 20 µM H_2_O_2_ for 2 h and then maintained in Neurobasal-A medium containing 50 µM EGCG with or without PKD1 inhibitor (CID755673)/ferroptosis inducer (erastin) for 48 h. Finally, cell viability, western blot, and ELISA assays were performed.


**Ethical approval::** The research related to animal use has been complied with all the relevant national regulations and institutional policies for the care and use of animals.

### 1.2 Cell viability assay

Cell viability assays were performed according to previous studies with minor modifications [38,39]. The Cell Counting Kit 8 (CCK-8; HY-K0301, MedChem Express, China) assay was used to evaluate cell viability. At the indicated time points, 10 µL of CCK-8 solution was added to each well in a 96-well culture plate, following a 2-h incubation at 37°C. Then, absorbance was measured in a multiwell plate reader (Tecan Infinite^®^ M1000 Pro) at 490 nm.

### 1.3 Enzyme-linked immunosorbent assay (ELISA)

ELISA was performed according to previous studies with minor modifications [40]. ELISA kits manufactured by Hanhong Biochemical Company (Sino Biological, China) were used to measure PKD1 phosphorylation and related ferroptosis marker activities. Absorbance at 450 nm was measured by an ELISA reader (Tecan Infinite® M1000 Pro).

### 1.4 Surgical procedures for complete ST

The surgical procedures were performed on female rats according to previous studies with minor modifications [39]. Briefly, the T9 segment of the spinal cord was completely removed using angled microscissors. After confirming the completed transection by lifting the cut ends of the cord, the surgical incisions were sutured. Following surgery, animals were placed on a heating pad to facilitate their recovery from anesthesia and surgery and then housed in individual cages. All animals survived for 2 months after the surgery.

To investigate the neuroprotective role of EGCG after complete ST, eight rats per group were randomly divided into four groups: (A) ST, (B) ST + EGCG, (C) ST + EGCG + CID755673 (PKD1 inhibitor), and (D) ST + EGCG + erastin (ferroptosis inducer). The treatment groups were intraperitoneally injected with 100 µL of phosphate-buffered saline or EGCG (the final concentration diluted in blood was 50 µM) with or without CID755673/erastin once daily for 7 days after the surgery. The rats without ST or EGCG treatment were used as the Sham control. No animals died during the 12-week survival study, after which the animals were sacrificed.

### 1.5 Behavioral tests

Hindlimb locomotor function was investigated by the Basso, Beattie, and Bresnahan (BBB) locomotor rating scale, inclined grid climbing assessment and rotarod test.

BBB scoring was performed as previously described [39,41], and the rats were placed in an open field (70 cm × 70 cm × 50 cm) to move freely.

The grid climbing test was performed as previously described [39,42]. Animals had to move from the bottom to the top of a grid placed 45° from the horizontal plane. Videos were recorded for more than 5 min each. The knee joint angle of animals climbing the grid and the number of animals whose hindlimbs gripped the grid during climbing were calculated.

The rotarod test was performed as previously described [43]. The Rotamex Rotarod system (UGO Basile Rat&Mouse Rota-Rod 47700/600, Italy) contained a rotating rod that was divided into five parts by a splitter plate to permit the testing of five animals at one time. The test was performed in the same mode, and the speed when the rat fell off was recorded. Each animal was tested three times to obtain the average value.

### 1.6 Tissue processing

At 8 weeks after the surgery, the animals were euthanized by deep anesthesia with isoflurane and transcardially perfused with saline. Then, the L3 segment of the spinal cord and biceps femoris muscle was dissected and harvested for further analyses.

### 1.7 Western blot analysis

Equivalent quantities of cell lysates were combined with 20% loading buffer (LB) (0.125 mol/L Tris–HCl, pH 6.8, 20% glycerol, 10% sodium dodecyl sulfate, 0.1% bromophenol blue and 5% β-mercaptoethanol) and heated at 95°C for 15 min. Samples were resolved by 10% sodium dodecyl sulfate-polyacrylamide gel electrophoresis and electroblotted onto polyvinylidene difluoride membranes (Millipore, Billerica, MA, USA). Nonspecific protein binding sites were blocked with 5% nonfat milk or 5% bovine serum albumin (BSA) diluted in Tris–HCl saline buffer containing 0.1% Tween-20 (TBST, pH 7.4). Membranes were incubated with the specific antibodies rabbit anti-pErk1/2 (1:1,000, ab4370; Abcam), rabbit anti-Erk1/2 (1:1,000, ab4695; Abcam), and anti-GAPDH (1:1,000; Beyotime Biotechnology) overnight at 4°C. After three washes with 0.1% TBST for 5 min, a horseradish peroxidase-conjugated goat anti-mouse secondary antibody (1:1,000; Boster) diluted in TBST was added, followed by three washes with 0.1% TBST for 5 min each at room temperature. Antigens were visualized using enhanced chemiluminescence (Beyotime Biotechnology). The intensity of immunostaining was measured with ImageJ 6.0 software.

### 1.8 Statistics

All statistical analyses were performed using GraphPad Prism 6 software. Data are reported as mean ± standard deviation and were analyzed using analysis of variance followed by the post hoc Bonferroni test. *p* < 0.05 was considered to be statistically significant.

## 2 Results

### 2.1 EGCG increases the survival rate and upregulates PKD1 phosphorylation in a dose-dependent manner

To evaluate the effect of EGCG on CGNs, cell viability and ELISA assays were performed after the CGNs were treated with various concentrations of EGCG (0, 0.5, 5, 50, and 500 µM) for 48 h.

**Figure 1:**
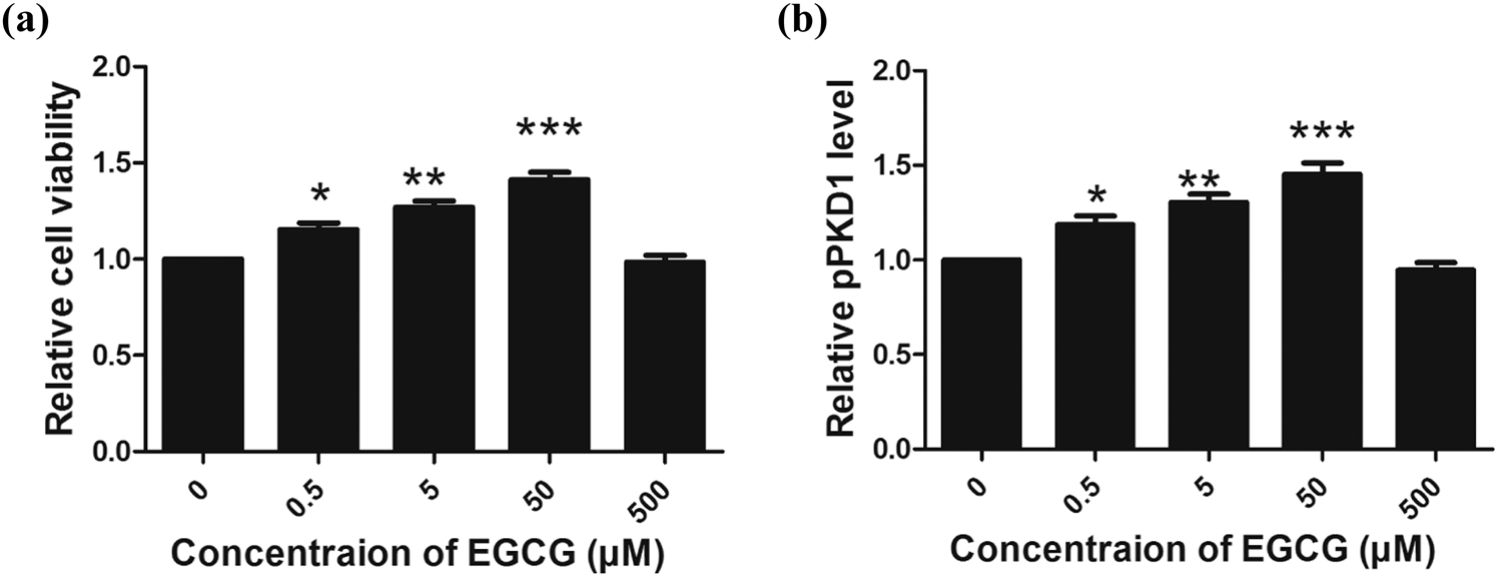
Effect of EGCG on cell viability and PKD1 phosphorylation in CGNs *in vitro*. (a) EGCG increased the peak levels of CGN survival and PKD1 phosphorylation at a concentration of 50 µM (a and b). (*p < 0.05, **p < 0.01, ***p < 0.0001, five independent experiments.)

We observed that the survival rate of CGNs was increased in response to EGCG treatment in a dose-dependent manner, with a peak level observed at a concentration of 50 µM (Figure 1a). A similar pattern for PKD1 phosphorylation levels was also observed (Figure 1b).

These results revealed that EGCG treatment at a concentration of 50 µM can increase the CGN cell survival rate with a maximum effect. As a consequence, 50 µM, the maximum protective concentration of EGCG (also observed in our previous study [44]) was selected to investigate the neuroprotective role of EGCG in the following *in vitro* and *in vivo* models.

### 2.2 EGCG upregulates PKD1 phosphorylation to inhibit ferroptosis and increase the survival rate of CGNs under oxidative stress

To evaluate the effect of EGCG on the survival of CGNs under oxidative stress, cell viability and western blotting were performed after CGNs were treated with 20 µM H_2_O_2_ for 2 h and following treatment with 50 µM EGCG or CID755673 for 48 h.

**Figure 2:**
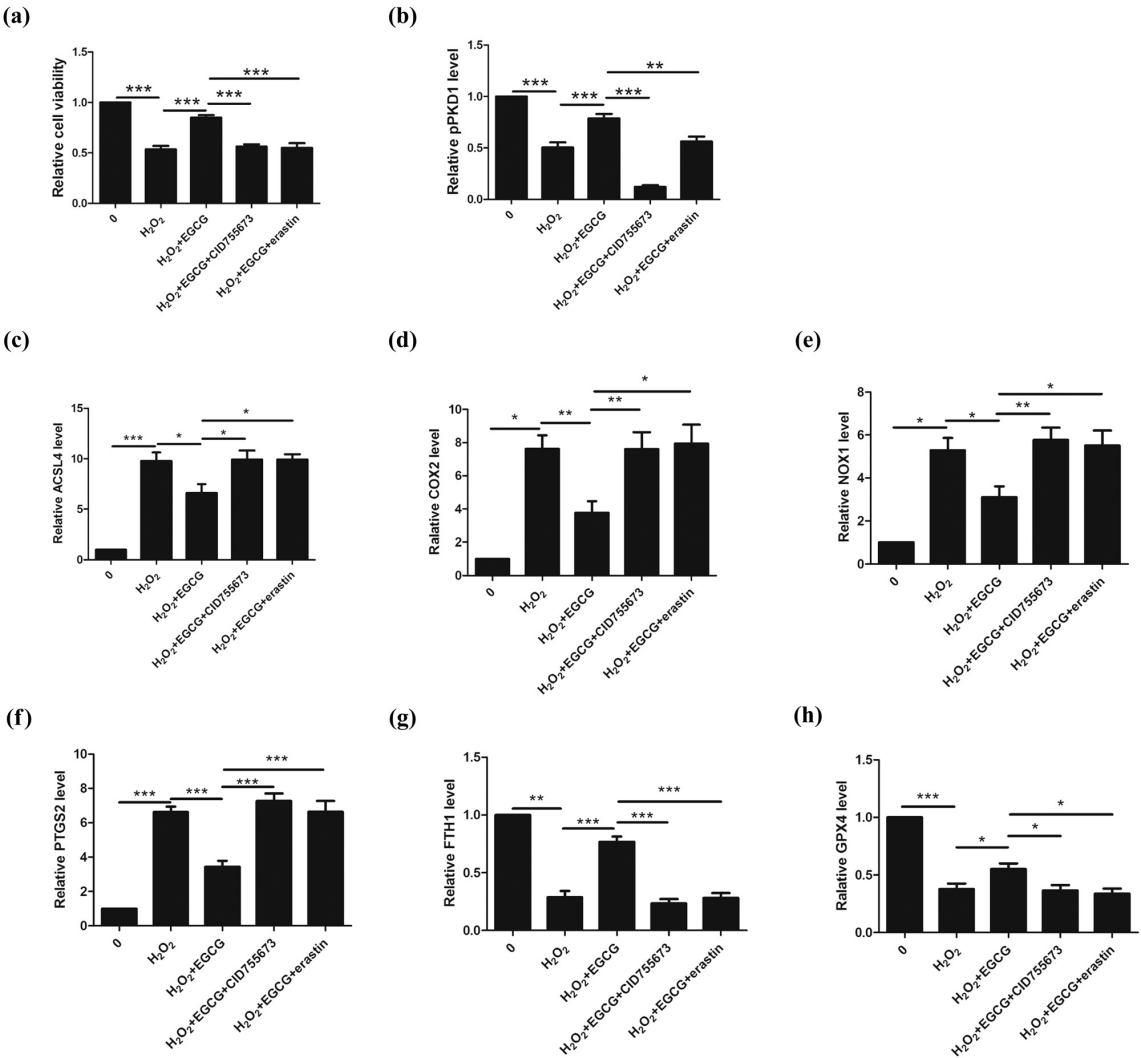
Effect of EGCG on CGN survival under oxidative stress *in vitro*. (a) EGCG protected against the cell death of CGNs induced by H_2_O_2_ by modulating PKD1 and ferroptosis. (b) PKD1 phosphorylation levels were increased in response to EGCG treatment in CGNs under oxidative stress. (c–h) Ferroptosis was inhibited in response to EGCG treatment in CGNs under oxidative stress, as indicated by the downregulation of ACSL4, COX2, NOX1, and PTGS2 and the upregulation of FTH1 and GPX4. (*p < 0.05, **p < 0.01, ***p < 0.0001, five independent experiments.)

We found that treatment with H_2_O_2_ decreased the cell survival rates of CGNs and PKD1 phosphorylation and promoted ferroptosis, whereas EGCG increased the cell survival of CGNs and PKD1 phosphorylation and inhibited ferroptosis induced by H_2_O_2_. When the cells were treated with CID755673, EGCG did not increase the cell survival of CGNs or inhibit ferroptosis induced by H_2_O_2_. After treating the cells with erastin, EGCG did not increase the cell survival of CGNs exposed to H_2_O_2_ (Figure 2a–h).

### 2.3 EGCG upregulates PKD1 phosphorylation to inhibit ferroptosis and promote the functional recovery of rats following ST

To investigate the effect of EGCG on the promotion of functional recovery in rats after complete ST, BBB scoring and grid climbing tests were performed.

**Figure 3:**
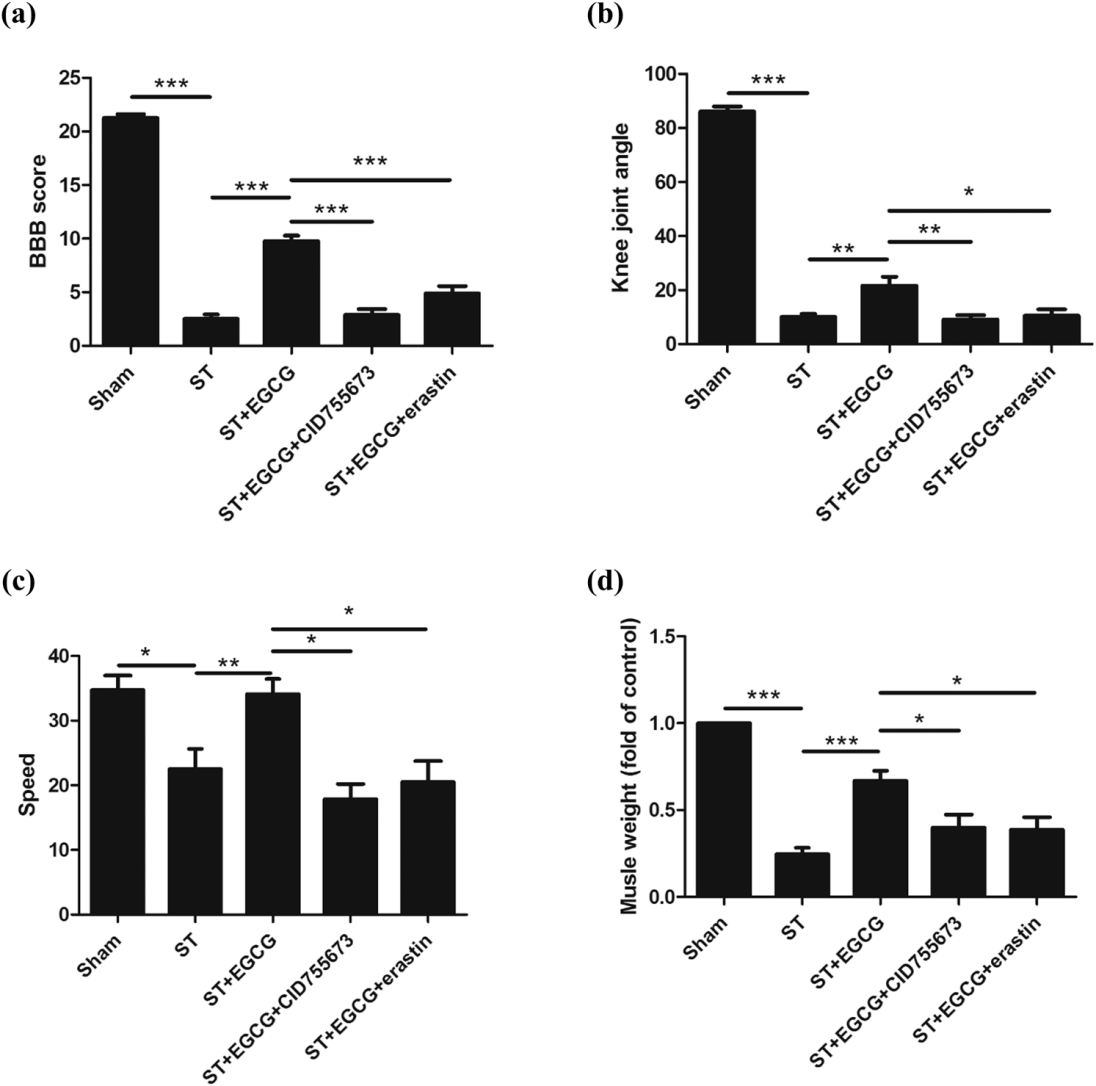
Effect of EGCG on CGNs and functional recovery in rats after complete ST. EGCG increased PKD1 phosphorylation and inhibited ferroptosis to promote functional recovery in rats, as indicated by (a) increased BBB score, (b) knee joint angle, (c) speed in the rotarod test, and (d) biceps femoris muscle weight. (*p < 0.05, **p < 0.01, ***p < 0.0001, *n* = 8.)

We observed that complete ST decreased the BBB score, whereas EGCG increased the BBB score after injury. In rats treated with CID755673, EGCG did not increase the BBB score after injury. After treating the rats with erastin, EGCG did not increase the BBB score (Figure 3a). Similar patterns for the knee joint angle and speed were also observed (Figure 3b and c).

We also observed that complete ST decreased the weight of the biceps femoris muscle, whereas EGCG increased the weight of the biceps femoris muscle. In rats treated with CID755673, EGCG did not increase the weight of the biceps femoris muscle after injury. After treating the rats with erastin, EGCG did not increase the weight of the biceps femoris muscle (Figure 3d).

### 2.4 EGCG upregulates PKD1 phosphorylation to inhibit ferroptosis and promote the survival of neurons in the spinal cord of rats following ST

To evaluate the effect of EGCG on the survival of neurons after complete ST, ELISA and western blotting were performed.

**Figure 4:**
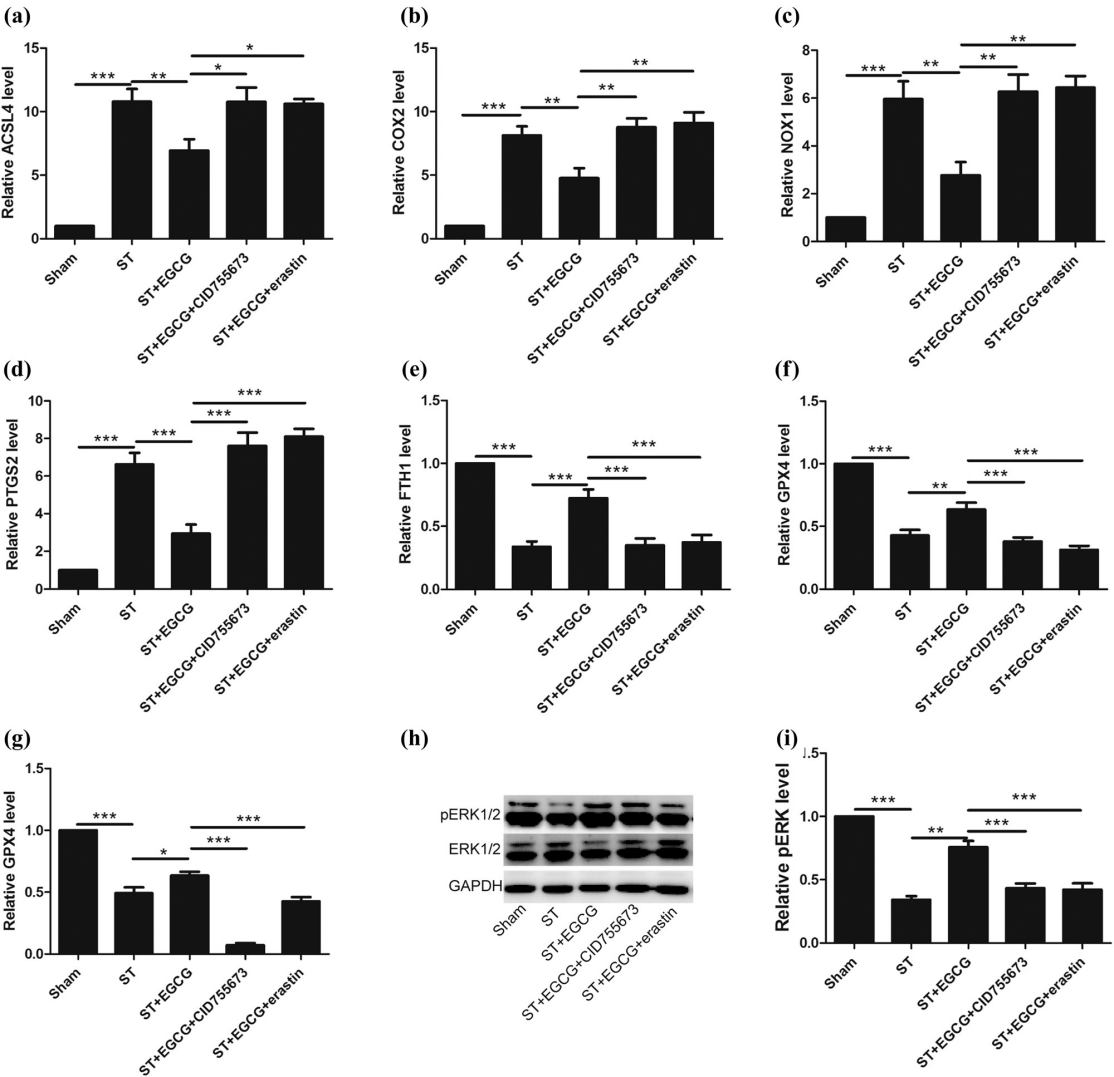
Effect of EGCG on neuronal survival in the spinal cord in rats after complete ST. (a–f) Ferroptosis was inhibited in response to EGCG treatment in the spinal cord, as indicated by the downregulation of ACSL4, COX2, NOX1, and PTGS2 and upregulation of FTH1 and GPX4. (g) PKD1 phosphorylation levels were increased in response to EGCG treatment. (h and i) ERK phosphorylation levels were increased in response to EGCG treatment. (*p < 0.05, **p < 0.01, ***p < 0.0001, *n* = 5.)

We found that ferroptosis was activated in response to complete ST, whereas EGCG inhibited ferroptosis in the spinal cord; moreover, EGCG could not inhibit ferroptosis when rats were treated with CID755673 and erastin (Figure 4a–f).

We found that PKD1 phosphorylation levels were downregulated in response to complete ST, whereas EGCG increased PKD1 phosphorylation levels in the spinal cord; moreover, EGCG did not increase PKD1 phosphorylation levels when CID755673 and erastin were used (Figure 4g).

We found that ERK phosphorylation levels were downregulated in response to complete ST, whereas EGCG increased ERK phosphorylation levels; moreover, EGCG could not upregulate pERK under injury in response to CID755673 and erastin (Figure 4h and i).

## 3 Discussion

Previous studies, including ours, have indicated the neuroprotective roles of EGCG in the nervous system [11,12,44]. In the present study, we revealed that EGCG increases neuronal survival and promotes functional recovery by modulating PKD1 phosphorylation and inhibiting ferroptosis under complete ST.

After SCI, the local microenvironment critically influences neuronal survival, axonal regeneration, and functional recovery [45,46]. Assessment of neurological function is a commonly used measure to evaluate the degree of injury and the therapeutic effect of medications [39]. In the present study, we observed that EGCG can promote functional recovery after injury.

Functional recovery can be spontaneously regained in SCI due to the survival of neurons in the spinal cord that control hindlimb locomotor activity below the lesion [47]. PKD1 has been reported to prevent neuronal cell death [24]. Moreover, PKD1 may function by regulating the ERK signaling pathway [36]. Previous studies have also shown that Erk1/2 signaling cascades exert a key role in the regulation of gene expression and prevention of apoptosis [48]. It has also been reported that the activation of MAPK/Erk can promote the differentiation and survival of neurons [49,50]. In the present study, we indicate that EGCG can increase PKD1 and ERK phosphorylation levels to promote neuronal survival after injury.

Therefore, about the question remains about what are the underlying mechanisms of EGCG to exert its neuroprotective effects. We then focused on the newly discovered type of cell death, ferroptosis, which requires simultaneous depletion of GSH or inactivation of GSH-dependent antioxidant enzyme GPX4 and incorporation of oxidizable polyunsaturated fatty acids into phospholipids [34]. GPX4, a lipid repair enzyme, is the central regulator of ferroptosis [32,33,51,52]. In the present study, we observed that EGCG can inhibit ferroptosis to promote neuronal survival after injury.

Taken together, these results indicate that treatment with EGCG partially accelerates functional recovery after complete ST by affecting PKD1 phosphorylation and inhibiting ferroptosis.

## Abbreviations

BBB: Basso, Beattie, and Bresnahan
CGNs: Cerebellar granule neurons
EGCG: (−)-Epigallocatechin-3-gallate
ELISA: Enzyme-linked immunosorbent assay
GPX4: Glutathione peroxidase 4
GSH: Glutathione
LB: Loading buffer
NF-κB: Nuclear factor-kappa B
PKD1: Protein kinase D1
ROS: Reactive oxygen species
SCI: Spinal cord injury
ST: Spinal cord transection.
